# Immunogenomic approaches to understand the function of immune disease variants

**DOI:** 10.1111/imm.12796

**Published:** 2017-08-10

**Authors:** Dafni A. Glinos, Blagoje Soskic, Gosia Trynka

**Affiliations:** ^1^ Wellcome Trust Sanger Institute Wellcome Genome Campus Hinxton Cambridge UK; ^2^ Open Targets Wellcome Genome Campus Hinxton Cambridge UK

**Keywords:** activation, autoimmunity, cell activation, genomics

## Abstract

Mapping hundreds of genetic variants through genome wide association studies provided an opportunity to gain insights into the pathobiology of immune‐mediated diseases. However, as most of the disease variants fall outside the gene coding sequences the functional interpretation of the exact role of the associated variants remains to be determined. The integration of disease‐associated variants with large scale genomic maps of cell‐type‐specific gene regulation at both chromatin and transcript levels deliver examples of functionally prioritized causal variants and genes. In particular, the enrichment of disease variants with histone marks can point towards the cell types most relevant to disease development. Furthermore, chromatin contact maps that link enhancers to promoter regions in a direct way allow the identification of genes that can be regulated by the disease variants. Candidate genes implicated with such approaches can be further examined through the correlation of gene expression with genotypes. Additionally, in the context of immune‐mediated diseases it is important to combine genomics with immunology approaches. Genotype correlations with the immune system as a whole, as well as with cellular responses to different stimuli, provide a valuable platform for understanding the functional impact of disease‐associated variants. The intersection of immunogenomic resources with disease‐associated variants paints a detailed picture of disease causal mechanisms. Here, we provide an overview of recent studies that combine these approaches to identify disease vulnerable pathways.

**Glossary**
Chromatin accessibility: a term describing regions of the genome free of nucleosomes, which might be a result of the sequence occupation by a DNA binding protein, such as transcription factors. The open chromatin regions, which can be assayed with **ATAC‐seq** (assay for transposase accessible chromatin followed by sequencing) or DNase‐seq, often have a functional role in regulating transcription and are enriched at promoters and enhancers.
**Chromatin immunoprecipitation followed by sequencing** (**ChIP‐seq**): an assay in which antibodies against an epitope of interest, such as a transcription factor or a histone modification, are used to pull down the protein of interest along with the genomic sequences interacting with it. The proteins are then washed off and the DNA is sequenced. The sequencing results in pile ups of reads concentrated at the positions of DNA ‐protein interaction, referred to as peaks.
**Genome‐wide association studies** (**GWAS**): a method that leverages the information from genetic variants spread through the whole genome and compares allele frequencies between a group of patients and a group of healthy controls, within the same population. If the frequency difference is statistically significant between the two groups (*P*‐value < 5 x 10^‐8^), the variant is reported as disease‐associated. GWAS can also be applied within a single group with quantitative measurements, such as a group of patients for which a response to a treatment is measured, or a group of healthy individuals for which blood cell counts have been recorded.
Hi‐C: a genome‐wide method that enables identification of chromatin conformation in three‐dimensional space.
**Linkage disequilibrium** (**LD**): a correlation between genetic variants, resulting from the non‐random inheritance of genetic regions.
**Quantitative trait locus** (**QTL**): a region of the genome that is correlated with a quantitative phenotype. Typically, genetic variants, such as single nucleotide polymorphisms (**SNPs**), are correlated with the gene expression levels (**eQTL**).

## Introduction

The primary role of the immune system is to protect the host from infection by a variety of pathogens constantly present in the environment. As such, genetic defects that cause loss of the immune system's activity result in recurrent infections and severe immunodeficiencies that are often life threatening. However, uncontrolled activation of immune cells may result in the response being targeted towards healthy cells causing tissue destruction and consequently autoimmunity.

It is estimated that one in five people suffer from at least one of the 81 documented autoimmune diseases in the USA.^1^ Despite the high prevalence, the molecular mechanisms that predispose to autoimmunity are not well understood. The clustering of autoimmune diseases in families has indicated a strong genetic component underlying pathological processes driving many complex immune‐mediated diseases. Early studies identified several loci with large effect sizes, including HLA‐DQB1 associated with type 1 diabetes (T1D),^2^ HLA‐DQ2 and HLA‐DQ8 associated with coeliac disease (CeD),^3^ and HLA‐DR4 associated with rheumatoid arthritis (RA).^4^ It is now well appreciated that most autoimmune diseases share a strong association with the MHC region.^5^

The susceptible genetic background of the HLA alone is often not sufficient to lead to the development of an immune‐mediated disease. For example, T1D, RA, multiple sclerosis (MS) and CeD, result from the combination of the risk genotypes of both the HLA and non‐HLA genes, as well as an environmental trigger. Genome‐wide association studies (GWAS) revealed that complex immune traits develop as a consequence of the interplay between hundreds to thousands of common variants^6^ with individually small effects on the overall disease phenotype. More than 497 susceptibility loci for autoimmune disorders have been identified,^7^ however, despite the large number of mapped variants, the heritability explained by the non‐HLA loci remains moderate. Even for the most successful examples such as MS, T1D or RA, where over a hundred risk variants have been mapped, the explained heritability varies between 20% for MS,^8^ 10% for T1D^9^ and about 5% for RA.^10^

A clear picture emerging from GWAS is that immune‐mediated diseases to some extent result from the dysregulation of the same biological pathways. For example, a locus encoding genes for receptors that control T‐cell activation, *CD28*,* ICOS* and *CTLA4*, is associated to CeD, RA and T1D. The sharing of genetic regions associated with immune diseases is widespread; of the 90 risk loci associated with T1D, RA, CeD and MS, 33 (37%) overlap between two or more diseases.^11^ However, the molecular mechanisms by which genetic variants hinder control of the immune system and cause autoimmunity have been determined for only a small number of variants. For example, missense mutations in the exon of the key immune regulator, *CTLA4,* induce severe immunodeficiency and a spectrum of autoimmune and autoinflammatory diseases.^12^ These rare monogenic disorders provide an insight into the control of the immune system in the presence of the dysfunctional genes. However, the same genotype‐to‐function logic cannot be easily applied to complex traits. First, associated loci map to regions of the genome with extended linkage disequilibrium (LD). The LD blocks often comprise tens to hundreds of highly correlated single nucleotide polymorphisms (SNPs). Therefore, in a statistical test, the high correlation between SNPs results in equivalent strength of the association signal spread throughout all of the variants in LD. Practically, this renders the variants indistinguishable from one another and hinders the prioritization of the causal variants based on the association statistics alone. Second, the majority of the associated variants localize to the non‐coding regions of the genome, implicating that the disease variants are likely to act through the dysregulation of gene expression. This poses a challenge because gene expression regulation can be cell‐type‐specific and therefore functional follow‐up studies have to be carried out in the cell types most relevant to the disease. However, for many immune diseases the exact pathological cell type is unknown. The final challenge is linking the associated SNPs to effector genes, as the causal variants may not necessarily affect the closest genes but instead act through long‐range genomic interactions.

**Figure 1:**
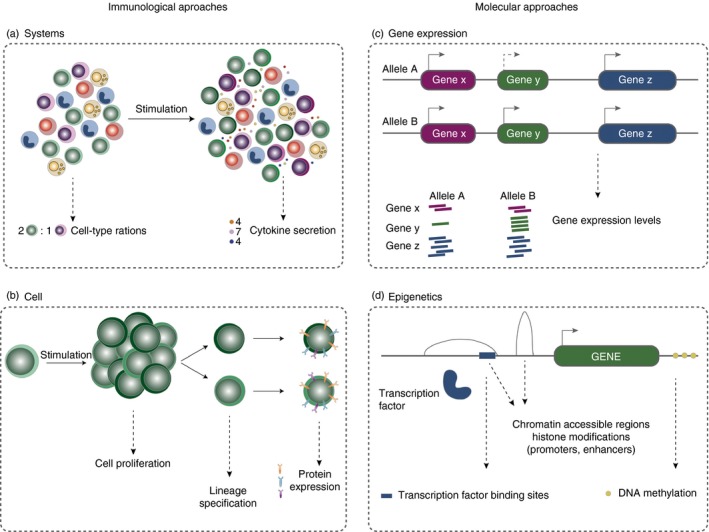
Immunogenomic approaches to infer the role of disease variants. Variants associated to immune diseases can be functionally annotated using immunological or a genomic approaches. Immunological approaches include the (a) systemic analysis of immune function, such as measuring cell type ratios and cytokine levels, and (b) cellular analysis, such as proliferation assays of a specific cell type in response to a stimulus, the lineage specification markers or protein expression levels. Genomic assays, include (c) gene expression and (d) epigenetics; transcription factor binding sites, chromatin accessibility, histone modifications and DNA methylation.

Here, we provide an overview of the recent advances in the genetics of immune‐mediated diseases. We discuss how the immunogenomic cross‐disciplinary toolkit can be used as a platform for inferring correlations between genotypes and individual genes, cellular traits, as well as the immune system as a whole (Fig. 1). Finally, we discuss the challenges of mapping associated variants to the molecular mechanisms through which they act.

### Correlating disease variants with molecular traits

#### Context‐specific effects of disease variants on gene expression

**Figure 2:**
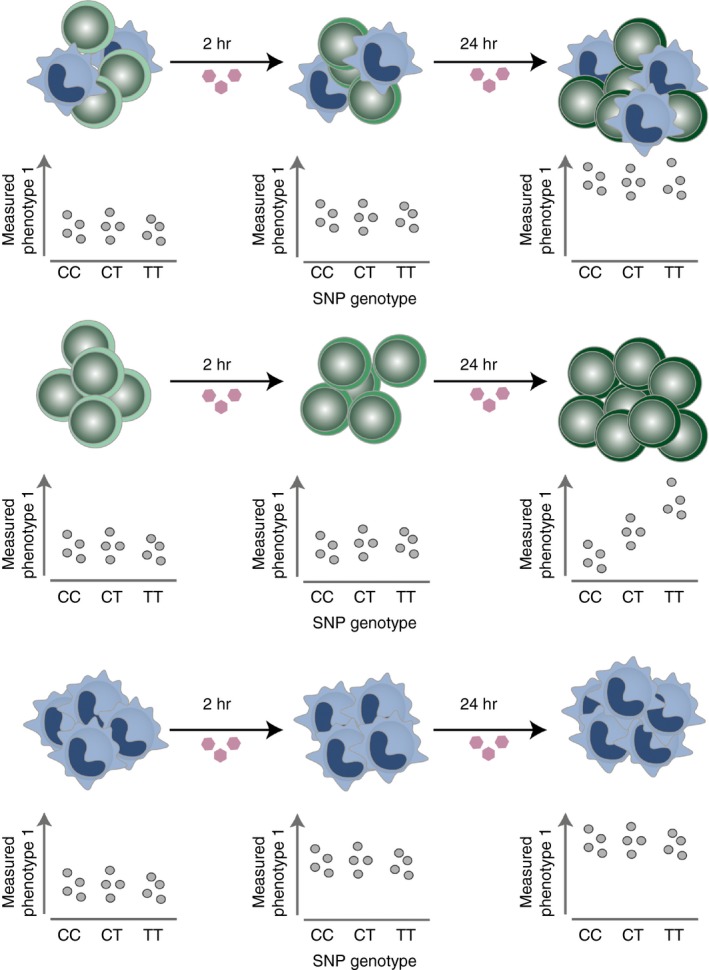
Cell‐type‐specific and cell‐state‐specific expression quantitative trait loci. Cellular phenotypes, such as gene expression, cytokine secretion or chromatin accessibility, might be affected by a genetic variant only in a specific cell type and under specific conditions, e.g. at a specific time‐point following a stimulation. Here, a bulk of cell types (upper panel), as well as each cell type individually, were stimulated for 2 and 24 hr. In all scenarios, the measured phenotype, e.g. gene expression, increased upon stimulation, but only in the green cell type (middle panel) was the effect correlated with the genotypes. The effect was missed when measured in the sample containing the mixed cell population (upper panel). The blue cell type (bottom panel) expresses the strongest up‐regulation in expression upon stimulation and largely drives the observed increase in expression in the bulk sample.

The effects of genetic variants on gene expression are often assessed through genotype correlation with gene expression levels measured across tens to thousands of individuals (expression quantitative trait loci, eQTL; Fig. 2). Disease‐associated variants are enriched for eQTLs, suggesting that a large proportion of disease variants are likely to function through gene expression regulation.^13^, ^14^ A relevant and easily accessible tissue for immune diseases is blood. Early studies demonstrated that immune disease SNPs also affect gene expression in the whole blood or peripheral blood mononuclear cells. For example, over 50% of CeD variants also expressed an eQTL effect.^15^ Disease‐associated variants affecting gene expression can point towards a dysregulated specific pathway, e.g. five of the inflammatory bowel disease (IBD) risk variants were also eQTLs that increased the expression levels of *ITGA*,* ITGAL*,* ICAM* and *ITGB8* genes, encoding integrins, the pro‐inflammatory cell surface proteins that mediate leucocyte accumulation at the site of inflammation.^16^

As the gene expression studies increased in sample size,^17^ and expanded across different tissues^18^ and cell states,^19^ it became evident that the eQTL effects are widespread. For example, the study of whole blood gene expression from over 8000 individuals identified that nearly 6500 genes (44% of all tested genes) were under genetic control.^17^ However, importantly, recent studies have highlighted that the initially observed over‐representation of GWAS SNPs among eQTLs may have been overestimated. This is because of the confounding effects of the LD and cell‐type‐specific gene expression. The LD results in long‐distance correlations between tens to hundreds of variants. The GWAS and eQTL signals can overlap in genetic location; however, it is critical to determine whether the overlap is coincidental or driven by the same functional variants. Therefore, simple overlap between the eQTL and the GWAS SNPs is not sufficient, instead other more stringent methods that co‐localize the LD variants between the two signals need to be applied.^20^, ^21^, ^22^ Guo *et al*.^20^ assessed the co‐localization of 595 variants from 154 non‐overlapping regions associated with ten different immune‐mediated diseases with gene expression variants from primary resting and stimulated monocytes as well as resting B cells. Of the 1414 genes mapping to these regions 125 showed an eQTL effect that also overlapped with a disease SNP. However, for only six genes was there a strong support of co‐localizing signals.

The very early eQTL mapping studies have already recognized the importance of cell‐type‐specific gene regulation. Dimas *et al*.^13^ used lymphoblastoid cell lines, primary fibroblasts and T cells from umbilical cords and reported that 69–80% of regulatory variants affected gene expression in a specific cell context. Indeed, a recent study found that only a small proportion of IBD variants overlap with whole blood eQTL SNPs (8 of 76 IBD loci).^23^ The lack of enrichment could be due to the presence of heterogeneous populations of immune cells in the whole blood. In fact, a higher enrichment was observed with eQTLs from CD4^+^, ileum and CD14^+^ cells, underscoring the importance of the cell‐type‐specific context when assessing the function of GWAS variants. Kasela *et al*.^24^ assessed gene expression in CD4^+^ and CD8^+^ T cells from 313 individuals and observed that the protective allele associated with T1D, a missense variant in *IL27*, decreased expression levels of *IRF1*,* STAT1* and *REC8* specifically in CD4^+^ T cells. Additionally, the expression levels of *IL27RA* and *IL6ST* genes, which together comprise the *IL27* receptor, were higher in CD4^+^ T cells in comparison to CD8^+^ T cells. This implies that a decreased level of interleukin‐27 (IL‐27) receptor in CD4^+^ T‐cell pathways could play a protective role for T1D.

Regulatory variants can exhibit opposite effects across different cell types.^25^ For example, of over 7000 eQTLs that were shared between monocytes and T cells, Raj *et al*.^26^ identified 42 eQTLs as having inverse effects between the two cell types. One of these eQTLs affected the expression of *CD52*, a target for antibody therapy used in MS treatment.^27^ Furthermore, even closely related cell types, such as CD4^+^ and CD8^+^ T cells, showed a limited overlap between eQTLs and GWAS SNPs (21 SNPs affecting the expression of 133 genes) based on a cohort of 313 healthy individuals.^24^ Better understanding of disease‐associated variants in a cell‐type‐specific context can therefore inform future drug development strategies, for instance by ensuring that a drug is targeting a protein within a specific, disease pathological cell type.

However, even if the relevant cell type is identified, the functional effect of a variant may not be detected unless the cell is challenged in an appropriate environment. Fairfax *et al*.,^19^ stimulated monocytes with lipopolysaccharide and interferon‐*γ* and demonstrated that 467 eQTLs overlapped with disease‐associated GWAS SNPs, 53% of which were stimulation specific. One of the eQTL‐GWAS SNPs was an MS variant that affects *IRF8* expression following 2 hr stimulation with lipopolysaccharide. Earlier studies that investigated the expression levels of *IRF8* in peripheral blood mononuclear cells using microarrays failed to identify a variant controlling the expression of this gene,^28^ probably due to the cell‐type‐specific effect. The same locus is also associated with SLE but with an opposite effect. *IRF8* is a regulatory factor of type‐1 interferons, which are elevated in patients with SLE, whereas patients with MS present low interferon levels.^29^ Importantly, the authors observed eQTL effects that differed between early and late stimulatory responses. Similarly, the dynamic nature of gene expression regulation was also observed in dendritic cells stimulated with interferon‐*γ*,^30^ and in CD4^+^ T cells stimulated with anti‐CD3/anti‐CD28 beads.^31^ Ye *et al*. identified 157 GWAS SNPs that overlapped with genetic variants that affected gene expression in CD4^+^ T cells in a cohort of 348 healthy individuals.^31^ Notably, an ulcerative colitis variant nearby *IL23R* and a variant nearby *IL2RA* associated with T1D, MS and vitiligo, only presented an effect on gene expression 48 hr following stimulation. Given that most effector functions of immune cells are performed following stimulation, it is not surprising that the majority of immune disease variants are functional in activated cells. Further studies are necessary to investigate different stimulation contexts in more detail.

#### Co‐localization of SNPs with chromatin marks points to disease causal cell types

Gene expression regulation results from the tight interplay between gene enhancers and promoters. Chromatin immunoprecipitation followed by sequencing (ChIP‐seq), assesses DNA–protein interactions by pulling down regions of the genome that are bound by the protein of interest. Recently, this method has been used to annotate the activity of non‐coding regions of the genome through the presence of different post‐translational histone modifications. Histone marks highlight genomic regions that regulate gene expression, they reflect change in chromatin structure that is often coupled with DNA accessibility for binding by different proteins, such as transcription factors (TFs). International consortia have worked together to comprehensively annotate the non‐coding sequences of the genome across a wide range of cell lines and primary cell types.^32^, ^33^, ^34^

**Figure 3:**
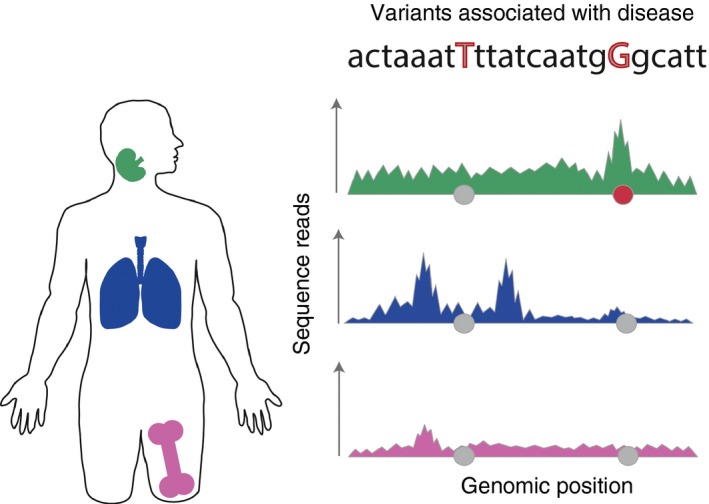
Causal disease variants overlap cell‐type‐specific chromatin marks. Chromatin immunoprecipitation followed by sequencing and chromatin accessibility assays produce pile ups of reads that form ‘peaks’. Such genomic annotations generated from different tissues [lymph nodes (green), lungs (blue), femur (pink)] provide a valuable roadmap of cell‐type‐specific genome activity. The genome annotations can be overlapped with genetic variants associated with a phenotype of interest, such as an autoimmune disease, represented as grey circles. If a statistically significant proportion of associated variants overlaps with peaks specific to a cell type it can point towards disease‐relevant tissue and prioritize the causal variants. Here, we illustrate a single‐associated locus where only one single nucleotide polymorphism (red circle) overlaps with a peak specific to the lymph nodes and absent from the other two tissues.

Comprehensive annotation of the regulatory chromatin provides an opportunity to interpret the role of non‐coding disease variants. Indeed, development of statistical approaches that integrate GWAS SNPs with histone marks proved to be a valuable approach to prioritize disease‐relevant cell types^35^, ^36^, ^37^ (Fig. 3). Enrichment of GWAS variants in cell‐type‐specific promoters marked by histone‐3 lysine‐4 trimethylation (H3K4me3), confirmed the importance of CD4^+^ T‐cell subsets in a number of autoimmune disorders, including T1D and RA. CD4^+^ memory T cells and regulatory T (Treg) cells showed high enrichment for variants associated with a number of diseases, including RA, IBD and CeD.^38^ This observation converges with the previous immune studies pointing towards impaired function of Treg cells in autoimmune diseases.^39^ Notably, a study by the Todd group has demonstrated that Treg cells from patients with SLE have lower levels of CD25, the *α*‐chain of IL‐2 receptor, which affects their phenotype and decreases their survival.^40^

Leveraging ChIP‐seq data allowed the mapping of a proportion of GWAS variants in active enhancer regions of CD4^+^ T‐cell subsets marked by histone‐3 lysine‐27 acetylation (H3K27ac).^37^ Consistent with eQTL data, stimulation‐induced enhancers had high enrichment for autoimmune disease GWAS SNPs, whereas no enrichment was observed in enhancers specific to resting cells.^37^ If performed across tens or hundreds of individuals, histone marks can be directly correlated with genotypes.^41^ That is because ChIP‐seq assays result in pile ups of sequence reads at genomic regions that map the interaction with proteins. In that respect, they produce a quantitative measurement and, just like gene expression, can be analysed in light of correlations with different alleles, as QTLs. Regulatory annotations are not only a good predictor of gene activity but can also suggest a mechanism of action, e.g. by implicating a specific TF binding site,^42^ or when overlapping with eQTLs.^43^ For example, histone acetylation QTLs in human lymphoblastoid cell lines are highly enriched for immune disease GWAS variants, particularly from MS. In a study of three major immune cell types; neutrophils, monocytes and CD4^+^ T cells, from 200 individuals, Chen *et al*.^44^ mapped coordinated genetic effects on the epigenome and transcriptome.

In addition to ChIP‐seq, genome activity can also be measured by chromatin accessibility, either through mapping regions that undergo cutting by DNase (DNase hypersensitive sites)^45^ or by incorporation of the Tn5 transposase (ATAC‐seq).^46^ Analysis of 349 tissues and cell types generated using DNase‐seq found that > 75% of non‐coding GWAS‐associated variants lie within the chromatin accessible sites. This implies that assaying chromatin activity provides a valuable approach for understanding molecular mechanisms underlying the disease variants. For example, around 25% (*n* = 262) of GWAS SNPs associated with autoimmune diseases residing within the DNase hypersensitive sites in immune cells altered the TF binding motif for the IRF9 pathway.^45^ The IRF9 network along with the Jak/Stat cascade are initiated in the presence of interferon‐*γ*, pointing towards a potentially important role for this pathway in the development of autoimmunity.

Despite the observed enrichment of disease variants in regulatory non‐coding sequences it is estimated that only 10–20% of 823 disease variants lie within transcription factor binding motifs, suggesting other mechanisms of gene expression regulation,^37^ for instance by affecting spatial genomic interactions. To test this, a promising approach includes an integration of GWAS variants with Hi‐C assay^47^ that infers chromosome conformation by mapping interactions between genomic regions located nearby in the three‐dimensional space. The technique allows both the high level mapping of chromatin loops as well as identification of more granular interactions between enhancers and gene promoters. The latter can be directly used in mapping non‐coding disease variants to target genes. For example, using Hi‐C, a recent study demonstrated that the non‐coding region on chromosome 6 containing a variant that has been associated with RA and psoriasis, interacts not only with the promoter of *TNFAIP3*, the closest gene, but also with *IL20RA*.^48^ Furthermore, a comprehensive examination of 17 human primary blood cell types found an enrichment of autoimmune disease SNPs in Hi‐C domains in lymphoid cells compared with myeloid cells.^49^ The authors identified that the majority of identified genes (76%) had not been previously linked with immune‐mediated diseases. Notably, five of the identified genes associated with RA and SLE were also eQTLs, e.g. *RASGRP1*, a gene that activates the extracellular signal‐regulated kinase/mitogen‐activated protein kinase cascade and regulates T‐cell and B‐cell development and differentiation.

Gene expression regulation is also reflected through DNA methylation. By combining DNA methylation with RNA sequencing from 3841 Dutch individuals, Bonder *et al*. observed that *trans* methylation QTLs, where genetic variants affect distant rather than local methylation status, were enriched for immune‐associated GWAS traits.^50^ Although methylation has typically been linked to repressed regions, the authors found two distinct functions for methylation depending on its location. To investigate these findings further, the authors carried out TF ChIP‐seq and identified 13 *trans*‐methylation QTLs that influenced the TF binding sites, including two SNPs on chromosome 4 associated with ulcerative colitis. They prioritized one of them as the causal SNP based on its association with lower methylation and the higher gene expression of *NFKB1*. By incorporating Hi‐C assay with DNA methylation and RNA sequencing, Bonder *et al*., showed that inter‐chromosomal contacts provide a mechanism by which some *trans*‐methylation QTLs can act. The 402 identified CpG islands overlapped with CTCF, RAD21 and SMC3 TF binding sites.

### Correlating genetic variation with immunological readouts

To gain comprehensive insights into the effects of genetic variants on the immune system, genomic assays have to be complemented with traditional immunological approaches. The network of cellular interactions can be assessed at a global scale, for instance by looking at mechanisms that control cell frequency and cell proliferation, as well as at the cellular level, by measuring protein expression and identifying genes that drive the phenotype of interest. To accurately infer the causal relationships between genetic variants and cellular traits it is important to carry out the experiments in cells isolated from healthy individuals, limiting the effects of active disease or ongoing treatment.

#### Linking GWAS variants to immune cell counts and function

The ratio between different immune cell subsets is heritable and could partly account for one of the pathobiological disease mechanisms.^51^, ^52^ Through an association study of genome‐wide SNPs with cell counts, two independent genetic signals were identified to control the CD4^+^ : CD8^+^ T‐cell ratio. Both signals mapped to the MHC region, explaining 8% of the observed variance. Interestingly, one of these associations overlapped with a T1D variant where the T1D risk allele increases the number of CD4^+^ cells by decreasing their apoptosis rate.^53^ Past years have seen many systematic, large‐scale efforts aiming to associate genetic variants with cell counts. Astle *et al*.^54^ identified over 2500 variants associated with 36 different haematopoietic traits. They observed an overlap between asthma‐associated variants and the eosinophil counts, highlighting that the established positive association between eosinophil counts and asthma is genetically controlled. Additionally, variants within the MHC locus and nearby *COG6*,* SPRED2*,* RUNX1* and *ATXN2/SH2B3/BRAP* genes pointed towards a novel link between eosinophil function and RA. Study of immune cell frequencies and surface protein expression levels of 170 dizygotic and 75 monozygotic pairs of twins using seven distinct 14‐plex antibody panels identified 151 independent heritable immune traits, including, for example, the proportion of CD10^+^ immature B cells associated with the genotypes of the *MME* gene, which encodes for a metallo‐peptidase.^55^ This study reported that one of the most heritable traits is the frequency of CD39^+^ Treg cells. The authors identified a SNP that increases the level of CD39, and so alters the proportion of CD39^+^ Treg cells. CD39 along with CD73 are enzymes that degrade the pro‐inflammatory ATP molecule to an anti‐inflammatory adenosine.^56^ Dysregulation of this machinery has been observed in MS, RA and IBD patients, with multiple drugs targeting these two proteins. Additionally, the same variant had previously been identified in a study that measured counts of 95 different cell types from a cohort of 1629 individuals from Sardinia.^57^

In another study, the authors demonstrated that genes located near RA‐associated variants were highly expressed in CD4^+^ effector memory T cells.^58^ Following this observation, Hu *et al*.^59^ isolated CD4^+^ T effector memory cells and measured relative cell abundance, as well as gene expression of 215 genes located in RA loci, and cell proliferation capacity upon *in vitro* stimulation with anti‐CD28/anti‐CD3. The authors identified a group of genes whose basal level could predict the proliferative potential of effector memory cells, along with a non‐coding genetic variant that increased cell division capacity. Although, this study has not linked RA risk loci with the effect on CD4^+^ memory cell proliferation, it exemplifies how to link genotype to immune cell function.

#### Genetic control of cytokine level in response to stimuli

The effector function of immune cells is to communicate with each other through expression of receptors and receptor ligands, combined with the secretion of specific molecules, such as cytokines. Alteration of these cellular functions results in an impaired immune response. Interestingly, the cytokine levels in the blood have been shown to be highly heritable.^52^ Systematic characterization of the cell response to different bacterial and fungal infections identified six cytokine QTLs (cQTLs) that explained the IL‐6, IL‐8, IL‐10 and tumour necrosis factor‐*α* levels.^60^ The genetic control of cytokine secretion in response to pathogens is relevant, as some of the identified cytokines have also been associated with autoimmune diseases and are being targeted by drugs, e.g. IL‐6 with RA and psoriasis.^61^ Interleukin‐6 pathways, along with IL‐1*β* were identified as being mostly driven by genetics, compared with environmental factors and the microbiome.^62^ Assessment of the responses of peripheral blood mononuclear cells, whole blood and monocyte‐derived macrophages to pathogenic and non‐microbial stimuli in 500 individuals identified cell‐type‐specific cQTLs, with monocyte‐specific cQTLs being associated with susceptibility to infectious diseases and T‐cell‐specific cQTLs being associated with autoimmune diseases. Separately, co‐localization of genetic variants that control cytokine levels with those that contribute to susceptibility to autoimmune diseases linked the vascular endothelial growth factor cascade with IBD and expression of the *α* subunit of the IL‐2 receptor with Crohn's disease and MS.^63^ By standardizing the blood culture of healthy volunteers and using flow cytometry analysis, Duffy *et al*., de‐convoluted complex immune response signatures to a range of stimuli.^64^, ^65^ They built a detailed reference of cytokine levels from healthy individuals and identified a group of donors that do not release IL‐1*α* upon stimulation, despite the presence of IL‐1*β*, suggesting a genetic component underlying this effect.^64^ Together these data demonstrated that the levels of a number of pro‐ and anti‐inflammatory cytokines are under genetic control. Future large‐scale characterization of the molecular mechanisms that control cytokine levels in health and disease will provide further insights into disease pathology.

## Discussion and future perspectives

Over the last decade, hundreds of immune disease loci have been successfully mapped. Recent advances in genomics have enabled functional follow‐up studies that provide insights into the molecular mechanisms by which risk variants drive disease pathology.

In this review, we have provided recent examples where the correlations of quantitative immunogenomic phenotypes with disease‐associated loci prioritized causal variants and suggested disrupted gene pathways and cellular functions. Although the majority of individual allelic effects have a miniscule effect on the overall phenotype, the effects can be higher on the molecular or cellular levels. Hence, future efforts have to be focused on identifying the critical disease cell types in which the associated variants are functional. These studies are challenging to conduct, as certain cell populations only represent a small proportion of the whole blood and need to be isolated using reliable markers and cell sorting. Importantly, isolated cell populations are often difficult to culture *in vitro*. This process has to be repeated a number of times on blood drawn from individuals of the same population to reach a cohort size large enough that it would allow for reliable identification of QTL effects. QTL effects have been annotated mostly in the context of expression assays, however, a proportion of GWAS variants may not act through gene regulation measured by bulk gene expression assays, calling for further development of genomic tools.

A challenge in GWAS follow‐up studies is that often the causal cell types are unknown. Typically, cell types have been identified through immunology studies where a limited set of specific cell markers, both intracellular and extracellular, have been used to characterize cell populations. A promising new method, that is unbiased to a predefined set of markers, is single‐cell RNA sequencing (scRNA‐seq). This approach can identify previously unknown heterogeneity in a sample based on gene expression measured at the individual cell level. There is now a major international effort that aims to use scRNA‐seq to characterize all human cells, the *Human Cell Atlas*.^66^ This resource will provide the most comprehensive annotation of gene expression, and when integrated with GWAS variants it will present a tremendous opportunity for the improved understanding of immune‐mediated diseases.

## Disclosure

The authors have no competing interests.
